# Invariance of molecular charge transport upon changes of extended molecule size and several related issues

**DOI:** 10.3762/bjnano.7.37

**Published:** 2016-03-11

**Authors:** Ioan Bâldea

**Affiliations:** 1 Theoretische Chemie, Universität Heidelberg, INF 229, 69120 Heidelberg, Germany; 2 Institute of Space Sciences, National Institute for Lasers, Plasma, and Radiation Physics, National Institute for Lasers, Plasma, and Radiation Physics, 077125, Bucharest-Măgurele, Romania

**Keywords:** extended Hückel method, Landauer formalism, molecular electronics, negative differential resistance, wide- and flat-band approximation

## Abstract

As a sanity test for the theoretical method employed, studies on (steady-state) charge transport through molecular devices usually confine themselves to check whether the method in question satisfies the charge conservation. Another important test of the theory’s correctness is to check that the computed current does not depend on the choice of the central region (also referred to as the “extended molecule”). This work addresses this issue and demonstrates that the relevant transport and transport-related properties are indeed invariant upon changing the size of the extended molecule, when the embedded molecule can be described within a general single-particle picture (namely, a second-quantized Hamiltonian bilinear in the creation and annihilation operators). It is also demonstrates that the invariance of nonequilibrium properties is exhibited by the exact results but not by those computed approximately within ubiquitous wide- and flat-band limits (WBL and FBL, respectively). To exemplify the limitations of the latter, the phenomenon of negative differential resistance (NDR) is considered. It is shown that the exactly computed current may exhibit a substantial NDR, while the NDR effect is absent or drastically suppressed within the WBL and FBL approximations. The analysis done in conjunction with the WBLs and FBLs reveals why general studies on nonequilibrium properties require a more elaborate theoretical than studies on linear response properties (e.g., ohmic conductance and thermopower) at zero temperature. Furthermore, examples are presented that demonstrate that treating parts of electrodes adjacent to the embedded molecule and the remaining semi-infinite electrodes at different levels of theory (which is exactly what most NEGF-DFT approaches do) is a procedure that yields spurious structures in nonlinear ranges of current–voltage curves.

## Introduction

Even restricted to the steady-state regime, studying charge transport through molecular devices is a difficult nonequilibrium problem, and the variety of methods to approach this problem utilized in the literature [1–4] may be taken as a manifestation of this difficulty. As a self-consistency test for the various approaches utilized [5–7], a check of whether the charge conservation condition is obeyed by the method in question is an aspect that has occasionally received consideration [2,4]. With a few exceptions [8–13], wherein the independence of the current of the position of the area traversed by current was also investigated, most studies of this kind only checked the fact that the current at the “left” and “right” ends of the molecule are equal [1,14–15]. Except (if at all) for simpler interface effects (e.g., those accounted for through ohmic contact resistances), conduction through macroscopic solids contacted to electrodes is determined by the properties of the solid itself, which are practically unaffected by the electrodes [16].

**Figure 1:**
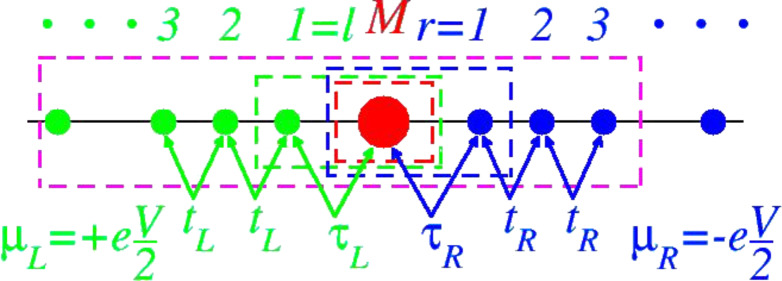
Schematic representation of molecular transport in a two-terminal setup consisting of a molecule (M) coupled to left (*L*→*l* = 1,2,3,…) and right (*R*→*r* = 1,2,3,…) electrodes under an applied bias *V*. In an adequate transport treatment, the current should be independent of how the system is partitioned, i.e., on how large the extended molecule (depicted by dashed rectangles) is.

Things drastically change in molecular junctions. There, upon contacting to infinite electrodes, the properties of the embedded molecule can be substantially modified with respect to the isolated molecule. This is particularly true in (chemisorption) cases where the anchoring groups form covalent bonds to the electrodes. Within current approaches to molecular charge transport, mostly based on nonequilibrium Keldysh Green’s functions (NEGF) combined with density functional theory (DFT), the molecular device is partitioned into a central region (also referred to as the “extended molecule”, “transport region”, “scattering region”, or “cluster”) linked to two semi-infinite “left” (L) and “right” (R) electrodes. This partitioning is inherently arbitrary. This arbitrariness is related to the arbitrariness in choosing the size of the extended molecule, which, in addition to the physical molecule, contains adjacent atomic layers (usually up to four) of (metallic) electrodes (for more specific details, see the subsection “Impact of screening and external biases”). An important problem related to this procedure is that metal atoms belonging to the extended molecule and metal atoms belonging to the electrodes are treated at different levels of theory. An unpleasant consequence is that this procedure may be a source of unphysical scattering for the electrons traveling from one electrode to another. The behavior, discussed later, is an illustration of such spurious effects. This may affect the theoretical current, yielding spurious structures (e.g., oscillations, shoulders or inflection points like those presented below) in computed *I*–*V* characteristics. A minimal mandatory requirement (“sanity test”) for any theory is whether the transport properties are independent of how this partitioning is made as shown in Figure 1. We are not aware of previous attempts in the literature to demonstrate or even check this invariance. Addressing this issue is one of the main aims of the present work.

## Results and Discussion

### General considerations

The considerations that follow refer to many-electron systems wherein correlation effects are negligible (i.e., the single-Slater determinant description applies). In such situations, the retarded Green’s function of the central region, **G***_C_*, linked to biased (*V* ≠ 0) electrodes is related to the retarded Green’s function of the isolated system, **G**_0,_*_C_* = (ε − **H***_C_*)^−1^ (i.e., uncoupled to electrodes) via the Dyson equation [1,4,16]:

[1]
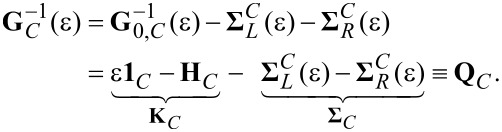


Here, **1***_C_* is the identity matrix, **H***_C_* is the Hamiltonian of the central region, and 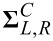 are embedding retarded self-energies, which characterize the coupling of the molecule to the left and right electrodes, respectively. Throughout this text, boldface symbols denote matrices in the electronic space of the central region (*C*). To simplify notation in the analysis that follows, orthonormal basis sets will be assumed throughout and matrices are referred to rather than operators. For nonorthonormal basis sets, the nondiagonal overlap matrix **S***_C_* would replace the identity matrix **1***_C_* of Equation 1. Switching between orthonormal and nonorthonormal basis sets leaves the diagonal matrix elements (on-site energies) unchanged while renormalizing the nondiagonal matrix elements (hopping integrals). See Chapter 4.1.2 of [4], which provides further details. Whenever possible, for convenience, the energy (ε measured from the equilibrium Fermi energy of the electrodes, *E*_F_ = 0) is omitted as well as other arguments of various functions entering the formulas given below. In the absence of correlations, the retarded Green’s function, **G***_C_*, remains, like in the equilibrium case, the key quantity allowing for the expression of all the relevant nonequilibrium properties. To illustrate this, the expressions of the transmission function, 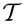, local spectral density of states, and charge densities are given below. The local densities of states (location indicated by μ) are given by the diagonal elements, 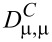, of the matrix **D***_C_*

[2]
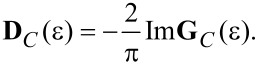


The charge densities *n*_μ_ are expressed by the diagonal elements 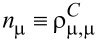 of the matrix 
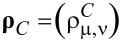
[16]:

[3]
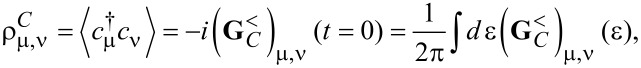


[4]
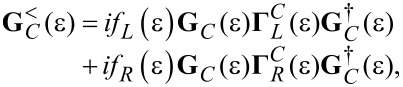


where *c*_μ_ and 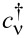 are electron annihilation and creation operators (entering the expression of the second quantized Hamiltonians, see below), and **G***^<^* is the so-called lesser Green’s function [16]. The transmission function is given by the trace formula [2,8,17]

[5]
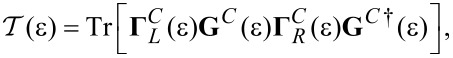


where the width functions

[6]
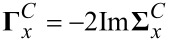


are determined by the imaginary parts of the retarded embedding self-energies 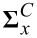, characterizing the molecule–electrode couplings (*x* = *L*,*R*). Within the Landauer approach, which provides a general framework to describe the molecular transport within the elastic, uncorrelated transport approximation, the (steady-state) current *I* through a molecular junction is obtained by integrating the transmission function 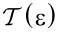
[2,8]

[7]
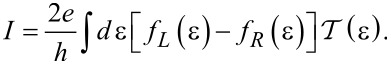


The difference between the Fermi distributions *f_L,R_*(ε) ≡ *f*(ε − µ*_L_*_,_*_R_*) of the biased (*V* ≠ 0) electrodes characterized by unbalanced Fermi energies μ*_L,R_* = ±*eV*/2 has an important role (albeit not the only one, see Figure 3 below) in determining the energy window of allowed (elastic) electron transfer processes contributing to the current. For simplicity, it is assumed that *V >* 0. Taking the limit *V*→0 in Equation 7, one gets the well-known formula for conductance, 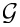, i.e., “conductance is transmission”:

[8]
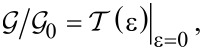


where 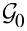 = 2*e*^2^/*h* is the conductance quantum. The second-quantized Hamiltonian of the molecular junction considered below, which is schematically depicted in Figure 1, reads

[9]
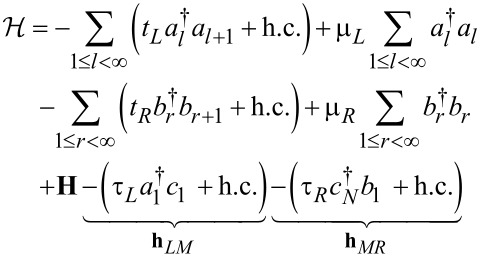


[10]
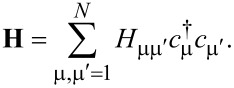


In the equations above, *a_l_* (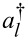), *b_r_* (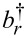), and *c*_μ_ (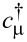) stand for annihilation (creation) operators for electrons in the left and right electrodes and in the “small” molecule, respectively. Restricting to spin-independent interactions for convenience, the spin label can be omitted and its contribution included by a factor of 2, like in Equation 7. For simplicity, the left and right electrodes will be modeled as semi-infinite chains described as a collection of sites (*l* and *r*, respectively), each site being characterized by a single level of site-independent energy μ*_L,R_* = ±*eV*/2 (remember that the equilibrium Fermi energy is chosen as origin *E*_F_ = 0) and nearest-neighbor hopping integral *t_x_* (*x* = *L*,*R*). The coupling between molecule and the left and right electrodes will be described in terms of the transfer integrals, τ*_L_* and τ*_R_*, which quantify the hopping between the left and right molecular ends (“anchoring groups”, denoted by labels 1 and *N*, respectively) and the adjacent electrode ends (labels *l* = 1 and *r* = 1 for left and right electrodes in Equation 9). In Equation 9, the small molecule, which needs not to be one dimensional, is described by a Hermitean Hamiltonian matrix, **H**≡ 
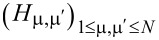
 = **H**^†^, whose elements (possibly complex numbers) are arbitrary.

Real systems described within the framework provided by Equation 9 and Equation 10 include atomic chains, quantum wires, carbon nanotubes, and (possibly DNA-based) bio- and larger organic molecules. For concrete cases, the model parameters (*H*_μ,μ′_, τ*_L_*_,_*_R_*, *t_L_*_,_*_R_*) can be obtained within density-functional-based, tight binding (DFTB) frameworks [18–20], which represent the state-of-the-art for such larger systems.

In a biased junction, they may also nontrivially depend on *V* and, if applicable, on gate potentials. In the description underlying Equation 9, the central region corresponds to the “small” molecule whose Hamiltonian is **H**. Equation 9 can be repartitioned by considering an extended molecule (Hamiltonian **H_ext_**) that includes parts of adjacent electrodes (*l*_0_ “layers” from the left electrode and *r*_0_ “layers” from the right electrode)

[11]
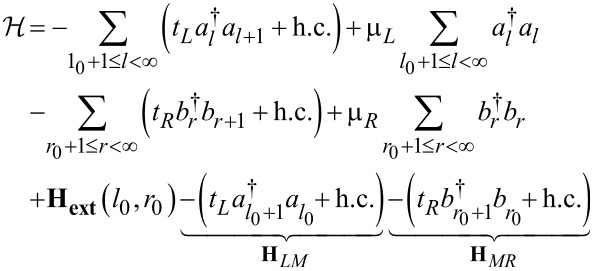


[12]
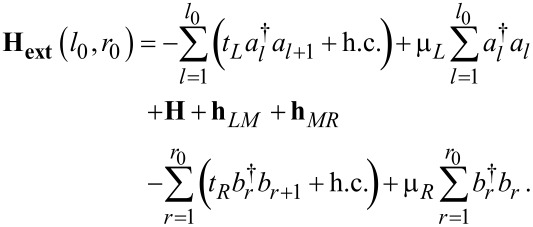


Whether using the small-molecule or the extended-molecule representation (i.e., Hamiltonians **H** or **H_ext_**(*l*_0_, *r*_0_), respectively), the electrodes are semi-infinite, homogeneous chains characterized by site-independent nearest-neighbor hopping integrals, *t_x_*, and on-site energies, μ*_x_*, (Equation 9 and Equation 11). In both aforementioned descriptions of the central region, the contact to each electrode consists of a single point, and the matrices 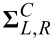 and **Γ***_L_*_,_*_R_* have a single, nonvanishing element. By choosing the small molecule as the central region, the embedding self-energy has the form

[13]
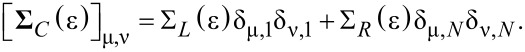


In the energy range of interest, the “surface” Green’s function, *g_x_*(ε), has the form [2,8,21]:

[14]
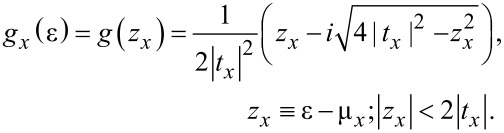


The embedding self-energy, 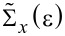, (a tilde is used for a generic, unspecified central region) can be obtained as

[15]
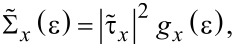


which via Equation 6 yields

[16]
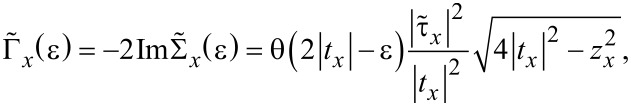


where θ is the Heaviside step function. Notice that the relevant energy range is |*z_x_*| = |ε − μ*_x_*| *<* 2|*t_x_*| since otherwise Γ*_x_* ≡ 0 and the contributions to Equation 2, Equation 4, and Equation 5 vanish. The quantity 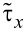 entering Equation 15 and Equation 16 is the hopping integral at the “interface” (in fact, the contact point) between the central region and electrode *x*. In the small molecule representation, 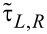 ≡ τ*_L,R_*. In the extended molecule representation, 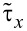 ≡ *t_x_* at the contact *x* where a certain number of electrode “layers” are included in the central region. The central region corresponding to the Hamiltonian operators expressed by Equation 9 and Equation 11 is different, namely, the “small” molecule (with Hamiltonian **H**) and the extended (“larger”) molecule (with Hamiltonian **H_ext_**), respectively. However, these two total Hamiltonians describe the same total physical systems and are mathematically identical. Therefore, whether computed within the description based on the small molecule or on an extended molecule, all the physical properties (e.g., transmission, current, local density of states, charge densities) should be identical. The proof that these properties indeed coincide, which represents an important sanity test for theory, will be given below.

For demonstration, a “minimally” extended molecule is considered, obtained by adding one extra electrode “layer” to the small molecule, namely the leftmost electrode site (*r* = 1) of the right electrode. This extended molecule having the Hamiltonian

[17]
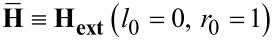


is schematically represented by the blue dashed rectangle in Figure 1. The demonstration goes as follows. We first show that the properties computed within the small molecule representation coincide with those based on the minimally extended molecule described above. Then, the demonstration for larger extended molecules follows immediately by induction. In the next step, the minimally extended molecule is taken as a new “small” molecule and choose the new extended molecule augmented with the next site of the right electrode. That is, **H_ext_**(*l*_0_ = 0, *r*_0_ = 1)→**H** and **H_ext_**(*l*_0_ = 0, *r*_0_ = 2)→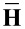. Notice that this is possible because, like **H** of Equation 10, the Hamiltonian **H_ext_**(*l*_0_ = 0, *r*_0_ = 1) is bilinear in the operators 
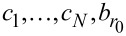
 and 
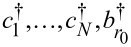
 pertaining to the corresponding “small” molecule. Obviously, this procedure can be reiterated by indefinitely pushing the right end of the extended molecule toward the right. Likewise, by interchanging the “left” and “right” labels one can gradually include (in addition to sites from the left electrode also) sites belonging to the right electrode (*l*_0_ = 1, 2,... in Equation 12).

### Demonstration that the representations based on the small molecule and minimally extended molecule yield identical physical properties

In the following, the quantities needed to compute the relevant properties are discussed for the cases where the small molecule and the minimally extended molecule are chosen as the central regions. All mathematical details and expressions needed for this explanation are given in Supporting Information File 1. Some key results are summarized in Table 1.

**Table 1:** Hopping integrals at the left and right contacts (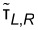), transmission function 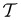, diagonal elements of the lesser Green’s function 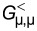, and the local density of states *D*_μ,μ_ for the cases where either the small molecule or the minimally extended molecule (as defined in the main text) are chosen to be the central region. The quantities in the same typeface (magenta, blue, and red) color are equal (cf. Equation 18, Equation 19, and Equation 20).

central region	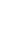	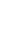	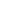	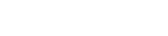	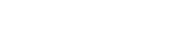

small molec.	τ*_L_*	τ*_R_*	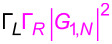	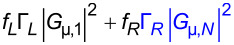	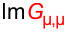
min. ext. molec.	τ*_L_*	*t_R_*	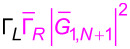	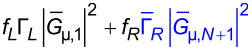	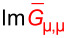

As developed in Supporting Information File 1 from Equations S4, S8, S12 and S14 (for η = 1)

[21]
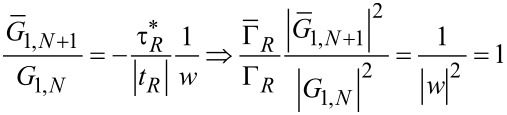


and therefore the ratio of the two transmission functions equals unity:

[18]
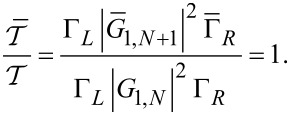


This fact is a direct consequence of Equation S11 in Supporting Information File 1. The equality of the local density of states computed in the small molecule and minimally extended molecule representation,

[20]
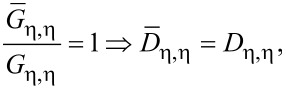


is the consequence of Equation S13a (for ξ = 1), Equation S13b (for μ = ν), Equation S4, Equation S8, and Equation S12 (1 ≤ η ≤ *N*) as defined in Supporting Information File 1. Equation S13a (for η = 1), S14, S4, S8, and S12 yield

[22]
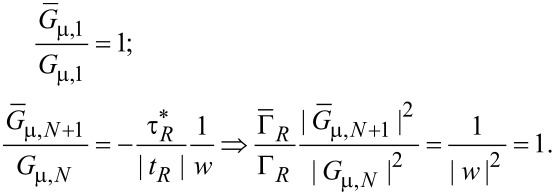


For the presently considered case, Equation 4 reads

[23]
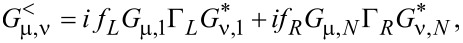


[24]
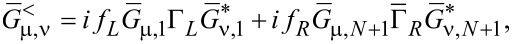


and then Equation 3 yields

[19]
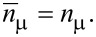


To sum up, Equation 18, Equation 20, and Equation 19 demonstrate that relevant nonequilibrium properties obtained within the small molecule and the minimally extended molecule representations coincide.

### Approximations of wide- and flat-electrode bands and related issues

At first sight, the concept of the invariance of the transport properties upon the choice of the extended molecule size may seem of merely academic interest (possibly a part of a Ph.D. tutorial) or useful for checking the correctness of numerical code to compute transport properties (which should not change whatever the size of the central region chosen). To see that the results presented above are also relevant for more pragmatic purposes, the effect of negative differential resistance (NDR) is discussed next in conjunction with common approximations used in transport approaches. In the preceding subsection, it is shown that the invariance of the transport is the direct consequence of Equations S10 and S11 from Supporting Information File 1, which follow from the exact expressions in Equation 15 and Equation 14. Whether they are also satisfied when approximate expressions are used instead of the exact ones will be discussed below. Here, the commonly employed limits of wide- and flat-electrode bands (labeled WBL and FBL, respectively) will be considered. Assuming embedding self-energies of the form (*x* = *L,R*),

[25]
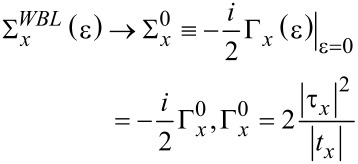


for all ε (wide-band limit, WBL), all energies in the Fermi window,

[26]
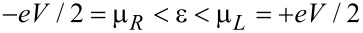


contribute to the integration entering Equation 7. This is an ubiquitous approximation not only in transport studies based on model Hamiltonians [1,4]; it is also an attractive approximation for realistic calculations (particularly for more reliably but computationally much more expensive approaches beyond NEGF-DFT treatments), as computation times can be radically reduced; see section “WBL-based schemes and realistic calculations” for more details. The restriction expressed by Equation 26 is imposed by the difference of the Fermi distributions, which are step functions at zero temperature, the case which is referred to below. The method based on Equation 25 and Equation 26, to which, as usual, is referred to as the wide-band limit (WBL), comprises in fact two approximations. It assumes (i) featureless (flat) electrode bands, characterized by energy-independent densities of states, and therefore Σ*_x_*(ε) is taken at the zero-bias Fermi energy ε = μ*_x_* = *E*_F_ = 0 and (ii) electrode bandwidths of ≈4|*t_L,R_*| much larger than any other characteristic energies (e.g., orbital energy offsets relative to the Fermi level and widths functions Γ*_L,R_*). This is the rationale to extend the integration over energy in Equation 7 up to infinity. Another possible approximation, which will be referred to as the flat-band limit (FBL) below, is to consider the energy-independent embedding self-energy, Σ*_x_*, given by Equation 25 but only in the finite energy range |*z_x_*| = |ε − μ*_x_*| *<* 2|*t_x_*|. The flat-band limit (FBL) is defined by Equation 27.

[27]
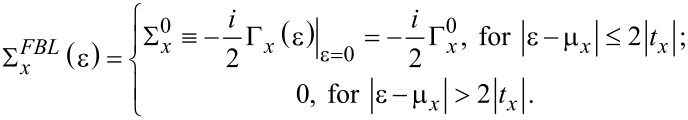


By taking ε = 0 and *V* = 0, Equations S10 and Equation S11 from Supporting Information File 1 are satisfied when the approximate expressions of Equation 25 and Equation 27 are employed. In this case, the approximate self-energies from Equation 25 and Equation 27 coincide with the exact one (Equation 15). Via Equation 8 and Equation 2 (see also Table 1), this implies that conductance and local density of states at equilibrium (*V* = 0) and zero temperature computed within the wide- or flat-band approximations are exact. Therefore, these quantities (as well as other properties corresponding to ε = *V* = 0) do not depend on the size of the central region. However, a straightforward inspection reveals that, in general (i.e., at arbitrary values of ε and *V*), Equations S10 and S11 from Supporting Information File 1 are no longer satisfied when the approximate expressions of Equation 25 and Equation 27 are employed. So, in general, the wide- and flat-band approximations do yield properties that depend on the size of the central region.

The following presents a further elaboration on this aspect, which is unphysical. In the illustrative examples presented below, the (small) molecule will be described as a single site (or level) of energy ε*_M_* whose Hamiltonian is given by

[28]
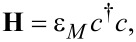


where *N* ≡ 1, *c* ≡ *c*_1_≡ *c_N_*, 
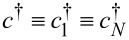
 in Equation 10. Notice that Equation 28 does not necessarily refer to a system consisting of a single site. It can describe a molecule wherein, as is often the case [22–24], the transport is dominated by a single molecular orbital.

#### Impact on the *I*–*V* curves

**Figure 2:**
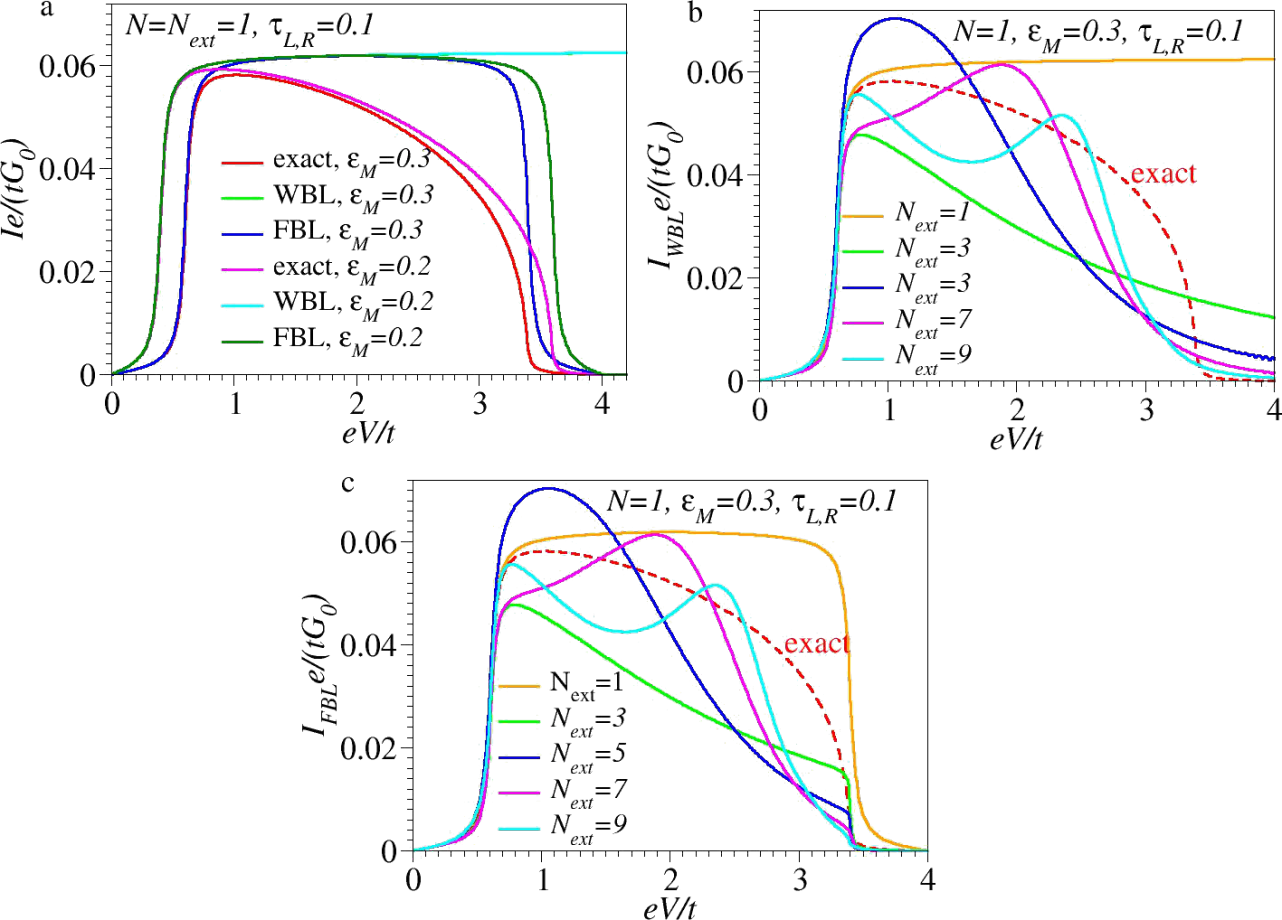
Dimensionless *I*–*V* curves computed for a small molecule consisting of a single site/level (*N* = 1) of energy ε*_M_*. (a) Exact results along with those obtained within the WBL and FBL, considering an extended molecule identical to the small molecule (*N* = *N*_ext_ = 1). (b,c) Exact results along with those obtained within the WBL (panel b) and FBL (panel c) using an extended molecule containing a number of sites *N*_ext_ = 1, 3, 5, 7, and 9. This mimics realistic calculations wherein up to four adjacent layers from electrodes are added to the small molecule. The energy unit is *t* = *t_L,R_* = 1, and the other model parameters are given in the inset.

**Figure 3:**
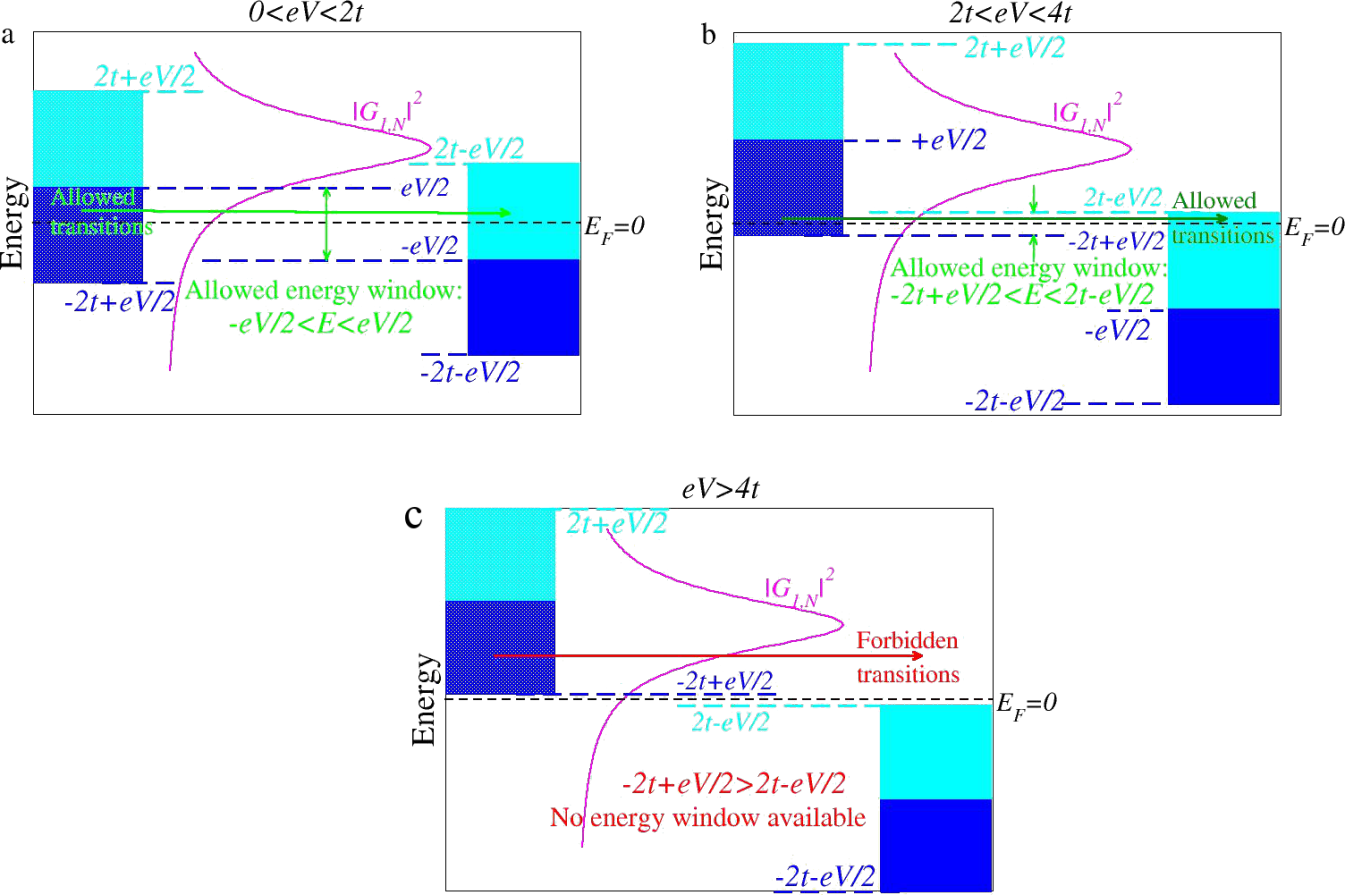
Schematic representation of the energy window available for elastic electron transitions at zero temperature (*t* = |*t_L_*| = |*t_R_*|, *V >* 0). (a) At biases 0 *< eV <* 2*t*, the (Fermi) energy window for allowed electron transitions becomes broader as the bias increases. (b) At biases 2*t < eV <* 4*t*, the energy window of allowed electron transitions (−2*t* + *eV*/2 *<* ε *<* 2*t* − *eV*/2) is narrower than the Fermi energy window (−*eV*/2 *<* ε *< eV*/2); it becomes narrower as the bias increases. This is reflected in a current decrease, i.e., a negative resistance behavior. (c) The current vanishes for *eV >* 4*t*, a bias range wherein there are no allowed electron transitions.

Figure 2 depicts various *I*–*V* curves computed for this case and various sizes of the extended molecule *N*_ext_ ≥ *N* = 1. While agreeing with the exact *I*–*V* curves at lower biases (*eV <* 2ε*_M_*), the approximate *I*–*V* curves significantly differ from the exact ones at higher biases. The wide-band approximation fails to describe the NDR regime exhibited by the exact *I*–*V* curves (Figure 2a). This is due to the fact that the width of the allowed energy window within the WBL (which is the Fermi energy window of Equation 26) contributing to the current (Equation 7) continuously increases with increasing *V >* 0. In fact, as depicted in Figure 3, this is true only for biases *eV <* 2*t*, as shown in Figure 3a. (To simplify the analysis, |*t_L_*| = |*t_R_*| = *t* is assumed.)

For biases 2*t < eV <* 4*t*, the width of the energy window of the allowed (elastic) transitions,

[29]
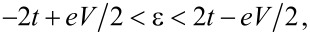


is narrower than that of the Fermi energy window (*eV*) determined by the difference of the Fermi distributions entering Equation 7. This is the straightforward consequence of the finite electrode bandwidths, as shown in Figure 3b. The fact that this energy width Δε = 2*t* − *V*/2 − (−2*t* + *V*/2) = 4*t* − *V* decreases with increasing *V* is reflected in a negative differential resistance (NDR) effect, which characterizes this regime. The physics underlying Equation 29 is correctly accounted for within the flat-band approximation (see the last line of Equation 27). This is why the description of the NDR effect is qualitatively correct within this approximation (cf. Figure 3b). Quantitatively, as visible in Figure 2, the FBL description of NDR is rather poor. This occurs because the energy window of the allowed transition (when correctly accounted for) is

**Figure 4:**
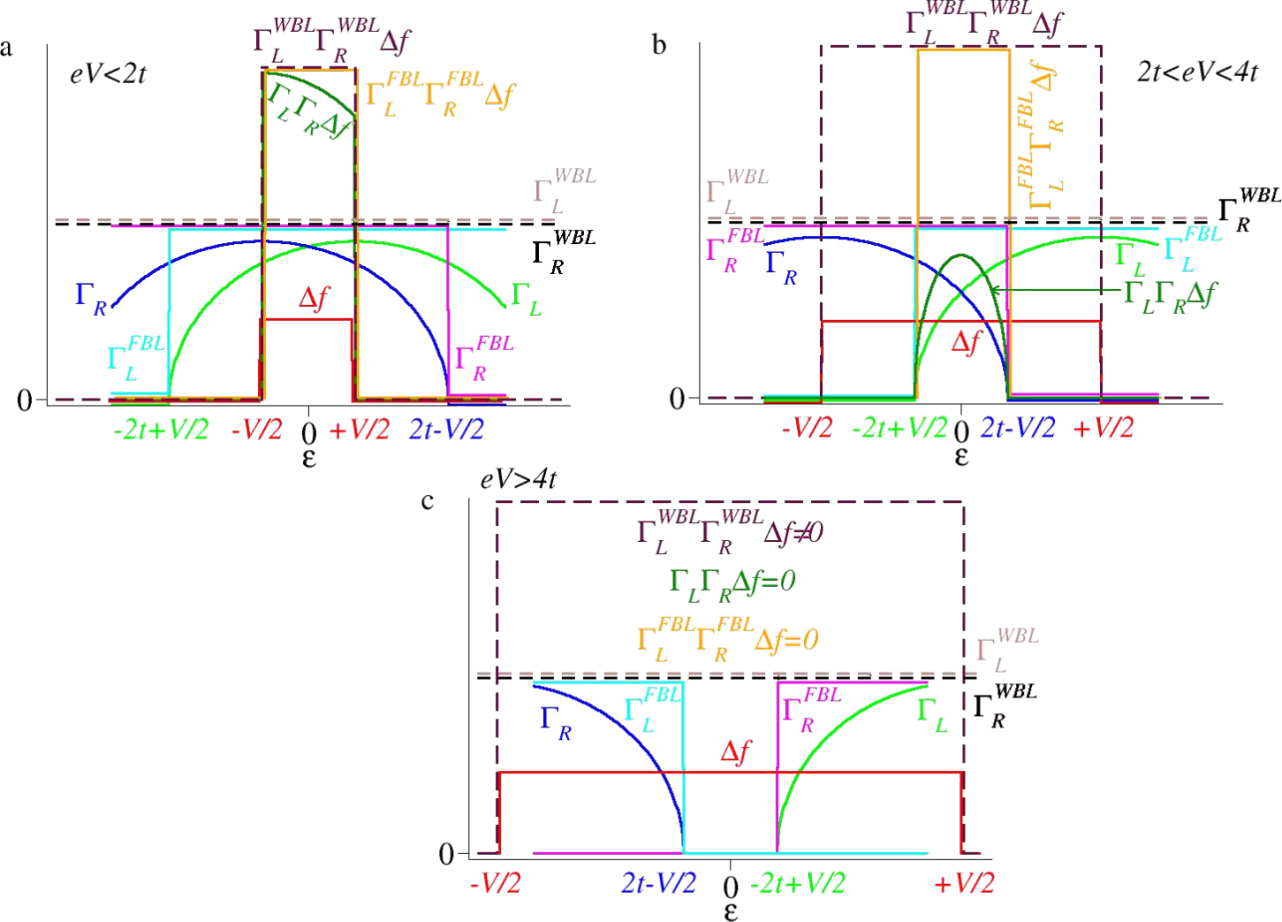
Schematic representation of the exact and approximate quantities entering the expression for the current in Equation 7 in the relevant bias ranges (0 *< eV <* 4*t*). Γ*_L,R_* are width functions computed exactly (no label) and within the wide-band and flat-band limits (labels WBL and FBL, respectively). Δ*f* ≡ *f_L_*− *f_R_* represents the difference between Fermi distributions of the left and right electrode. (a) At biases *eV <* 2*t*, the exact and approximate width functions behave similar (all are nonvanishing in the Fermi energy window −*eV*/2 *<* ε *< eV*/2 of allowed transitions), and this yields currents computed exactly and approximately, behaving qualitatively similar (cf. Figure 2). (b) The values for 2*t < eV <* 4*t*, Γ*_L,R_*, computed exactly and within the flat-band approximation, have nonvanishing values in an energy range narrower than the Fermi window, which becomes narrower as the bias increases. Therefore, the flat-band approximation can qualitatively predict an NDR effect. Still, because of substantial quantitative differences from the exact values, the FBL-estimated current is quantitatively unsatisfactory. (c) For *eV >* 4*t*, the energy ranges wherein Γ*_L_* and Γ*_R_* (computed exactly and within the FBL) have nonvanishing values that do not overlap. This yields vanishing currents, unlike within the WBL, which nonphysically predicts nonvanishing currents.

[30]
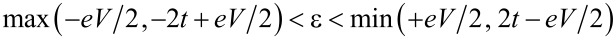


and not the Fermi energy window of Equation 26, and thus the energy dependence of the width functions Γ*_L,R_* is neglected within the FBL. As schematically shown in Figure 4, the ε-dependence of the width functions, which is weak at lower biases (cf. Figure 4a), becomes strong in the range defined by Equation 29, as shown in Figure 4b.

**Figure 5:**
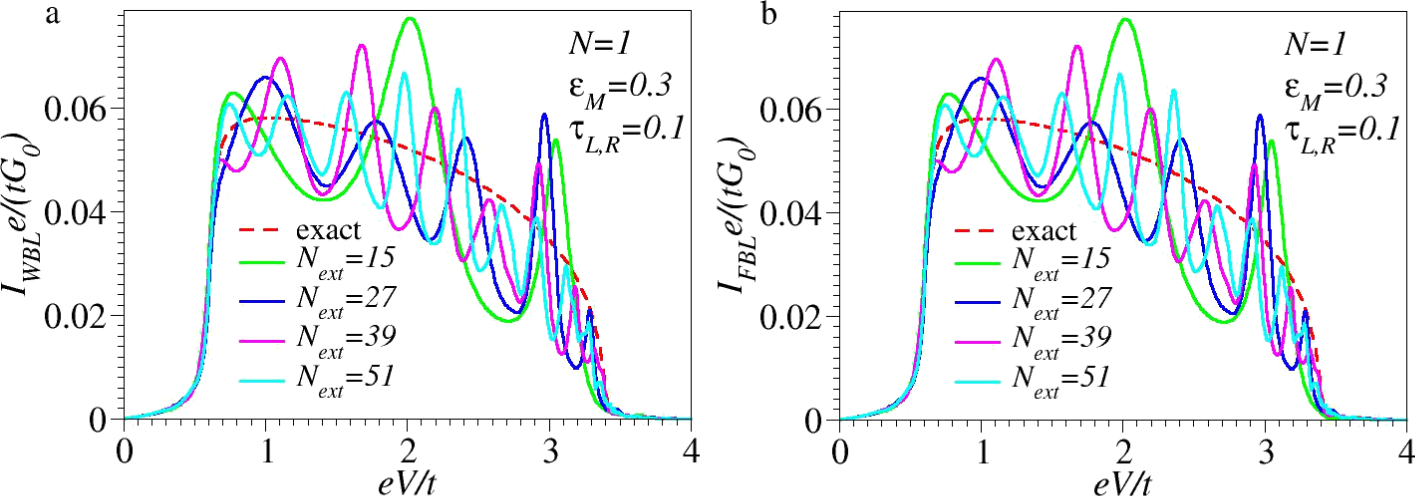
(a) Same as Figure 2b and (b) same as Figure 2c, but using extended molecule sizes up to *N*_ext_ = 51, that is, including up to 25 extra electrode layers at both sides, which is considerably more that present NEGF-DFT approaches can handle.

The fact that the NDR effect is overall underestimated within the FBL is due to the fact that the energy-dependence of Γ*_L,R_* is substantial (Equation 16). This yields a significant NDR even at biases *eV <* 2*t*, as revealed by the inspection of the exact *I*–*V* curves in the ranges 2|ε*_M_*| *< eV <* 2*t* (cf. Figure 2 and Figure 5). As illustrated by Figure 2a, there is no NDR within FBL for biases *eV <* 2*t*; in this bias range, the FBL and WBL results coincide (cf. Figure 3a, Equation 27 and Equation 25).

To complete this analysis, it is noted that the current vanishes for *eV >* 4*t* because there are no states available for elastic charge transfer processes (cf. Figure 3c and Figure 4c). While qualitatively this feature is correctly retained within the flat-band approximation, it is ignored within the wide-band approximation, which nonphysically predicts nonvanishing currents at these biases. Although the NDR effect per se is not the main focus of this paper, it is still noted that the NDR effect discussed above for a single site/level model is the consequence of the combined effect of the finite bandwidth and the energy dependence of the width functions (or, alternatively, the density of states at the contacts [21]). Electron correlations, which escape the conventional Landauer framework utilized here, can further enhance the NDR [25–27]. Besides finite bandwidth and energy-dependent densities of states, for systems with more than one site, the potential drop across the molecular bridge can be an additional source of NDR [28–29]. In accord with the general arguments presented above, Figure 2b,c illustrates that currents computed within the WBL and FBL, respectively, do depend (and significantly so) on the choice of the central region. The sizes utilized in these figures (up to *N*_ext_ = 9) mimic current NEGF-DFT transport calculations based on extended molecules including up to four adjacent layers from each electrode. Because the extended molecule considered in the present study is treated exactly, one may expect that by sufficiently increasing the size of the extended molecule, currents computed within the WBL and FBL would approach the values computed exactly. From this perspective, the results presented in Figure 5 are interesting. They show that even for extended molecules that are much larger than NEGF-DFT calculations can handle (given presently available computing resources), quantitative and qualitative deviations from the exact results for current beyond the ohmic regime are substantial.

The handful of examples presented above neither aimed at an exhaustive comparison of the exact results with those deduced within the wide- and flat-band approximations nor at a detailed discussion of NDR effects. They mainly aimed at demonstrating that in addition to the unphysical fact of breaking the invariance of the properties upon varying the size of the extended molecule, these approximations overlook significant physical effects. Even worse, as will be discuss next and anticipated in the Introduction, the WBL and FBL can predict spurious structures in the *I*–*V* curves at higher voltages. The examples depicted in Figure 5 fully support this idea: As visible there, the features exhibited by the WBL and FBL curves at high biases (oscillations, shoulders, inflection points), which have no counterpart on the exact curves, simply represent artifacts of inadequate approaches.

#### Impact on the charge distribution

**Figure 6:**
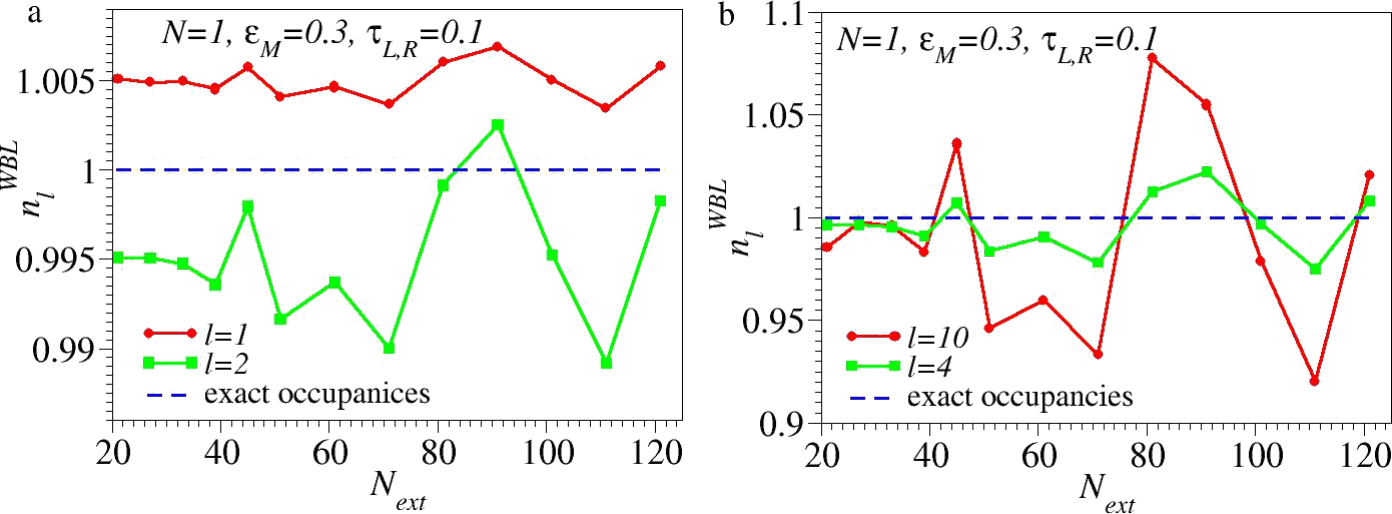
Even when increasing the size, *N*_ext_, of the extended molecule well beyond that which current computational facilities that employ NEGF-DFT implementations can handle (*N*_ext_ = 121 amounts to 60 electrode “layers” on either side), the occupancies 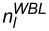 computed within the WBL do not approach the exactly computed values (the exact values represented by the blue horizontal lines differ from 1 by approx. 10^−5^). (a) Results for the first (*l* = 1) and second (*l* = 2) neighboring sites, and (b) fourth (*l* = 4) and tenth (*l* = 10) neighboring sites within electrodes relative to the “small” molecule, modeled as a single site and parameter values as given in Figure 2.

The unphysical dependence on the size of the extended molecule predicted by the aforementioned approximations does not only affect *I*–*V* curves and other nonequilibrium properties (which imply *V* ≠ 0), but also charge densities (or occupancies of molecular orbitals) at equilibrium (*V* = 0), to which energies other than the Fermi energy (ε = 0) contribute (cf. Equation 3) are affected. As a first example in this context one can mention the Friedel sum rule. The Friedel sum rule establishes an “exact” (see below why this word is put in quotation marks) relationship between two conceptually different quantities – ohmic conductance and level occupancy – for a nontrivial, single-level Hubbard–Anderson model. This model cannot be solved exactly in the general case because of the on-site Hubbard–Anderson interaction (*U* ≠ 0) between electrons of opposite spins occupying the same site/level, which is a source of (strong) electron correlations [30–33]. The approximation made to deduce this “exact” result (which also applies in the case *U* = 0, when the Hubbard–Anderson model reduces to the uncorrelated model described by Equation 28) is nothing but the wide-band approximation considered above. To see to what extent the “exact” Friedel sum rule is affected by the WBL one can compare the exact level occupancy with the occupancy obtained within the WBL. For the parameter values of Figure 2, the level occupancy estimated within the WBL deviates by 19% from the exact occupancy value. Although not dramatic, the error introduced by the WBL is still significant. As a second example, Figure 6 shows the occupancies 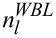 of several electrode sites at distance *l* from the embedded (small) molecule modeled as a single level/site computed within WBL using extended molecules up to very large sizes (*N*_ext_ ≤ 121, i.e., up to 60 “layers” in each electrode). While the small deviations from the exact values of occupancies of the electrode sites close to the (small) molecule (*l* = 1,2, Figure 6a) computed within WBL are acceptable, those for more distant sites (e.g., *l* = 4 and *l* = 10, Figure 6b) are unacceptably large. They amount to effective doping levels varying within up to approx. 10%, that is, they are comparable to the largest doping levels achieved experimentally in electronic devices of nanoscopic sizes [34–35]. These deviations of 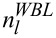 from the exact values, 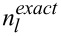, act as spurious charged scattering centers and are responsible for the artifacts in the *I*–*V* curves calculated within the WBL.

### Additional remarks

#### Impact of screening and external biases

As noted in the Introduction, the three-piece partitioning in an extended molecule linked to the left and right side of semi-infinite electrodes is a mental construct that is inherently arbitrary and should by no means affect the current and other physical properties. This is a minimal mandatory requirement for any valid theoretical approach. However, although conceptually arbitrary, for practical purposes, it is convenient that the partitioning fulfills certain conditions [36]. Unnecessary, more demanding computational effort can be avoided if the junction is partitioned such that: (i) the properties of the electrodes are homogeneous and do not differ from those of the bulk materials (metals), (ii) there is no direct interaction between the left and right electrodes, and (iii) interactions between the extended molecule and the two electrodes are merely confined to the extended molecule–electrode interfaces. “Screening” is the term under which conditions (i)–(iii) are usually listed in the context of realistic (DFT) calculations. The “extended” molecule should be taken large enough so that effects of the cluster to the (Kohn–Sham) potential outside the scattering region is screened. Outside the sufficiently large, extended molecule, screening effects should be altogether negligible, the potential should smoothly evolve into that of perfectly homogeneous bulk electrodes, and the charge distribution should match across the boundary of the scattering region and leads [37]. The inclusion of a sufficient number of additional electrode layers to satisfy these screening-related conditions may be an issue even for metallic electrodes and is a serious challenge for nonmetallic electrodes. One should note that the results presented in Figure 6, illustrating that even at sizes computationally prohibitive for microscopic studies, the charge density at the ends of the extended molecule computed within the WBL does not properly evolve into that of the bulk electrodes. The rather general model Hamiltonian of Equation 9 does satisfy these conditions. Condition (i) is satisfied because the electrodes’ parameters (on-site energies μ*_L,R_* and hopping integrals *t_L,R_*) are independent of the position (*l*,*r*). Condition (ii) is satisfied because 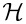 does not contain products (e.g., 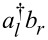) mixing operators from different electrodes. Condition (iii) is also satisfied because 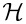 merely contains products involving nearest neighboring sites at the contacts, such as 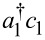 and 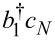 by choosing the small molecule described by the Hamiltonian **H** as central region, or 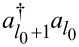 and 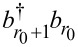 by choosing an extended molecule described by the Hamiltonian **H_ext_**(*l*_0_, *r*_0_) of Equation 12, which includes *l*_0_ “layers” of the left electrode and *r*_0_ “layers” of the right electrode. As discussed in subsection “Impact of screening and external biases”, in the model employed in this paper, all conditions for perfect screening are satisfied by using the “small” molecule as central region. Normally, to meet these conditions, several electrode layers are added to the “physical” molecule. For example, using the present nomenclature, the “small” molecule consists of a benzene dithiol (BDT = “physical”) molecule and several gold layers in the benchmark Au–BDT–Au junctions. For this reason, the term “small” molecule used in this paper refers to the smallest molecule (“smallest extended molecule”) satisfying the perfect screening conditions (i) to (iii) formulated in the text. The detailed demonstration exposed above clearly reveals that the invariance discussed in this paper also holds for situations wherein both the properties of the molecule and those of the interactions at molecule–electrode interfaces are affected by the applied bias *V*, gate potentials *V_G_*, defects (implying nonvanishing imaginary parts of the matrix elements):

[31]
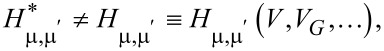


[32]
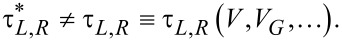


Although the electron spin has not been considered in order to simplify the presentation, the invariance discussed in this paper also holds in the presence of magnetic fields. One can see, for instance, that the quantities entering the relevant equations do not contain *t_L,R_* and 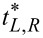 or τ*_L,R_* and 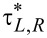 separately, but only |*t_L,R_*| and |τ*_L,R_*|. However complicated the dependencies in the right hand side of Equation 31 and Equation 32 might be, they do not break the invariance demonstrated above. So, this invariance holds regardless of the potential profile across the junction and how the (possibly very nontrivial) potential drop depends on the electrode–molecule contact interactions. This holds regardless of how involved the self-consistent procedures needed to determine the potential landscape are. Needless to say, these are all highly nontrivial tasks for realistic/DFT approaches, for which even the correct description of the both (positive and negative) bias polarities in simpler molecular junctions is an issue (see, e.g., the discussion related to Figure 5 in [22]).

#### WBL-based schemes and realistic calculations

Within all treatments of uncorrelated transport based on Equation 1 and Equation 5 (which is the case of all NEGF-DFT flavors), the WBL represents a computationally attractive approximation. The fact that the WBL embedding self-energies 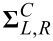 (hence also the width functions 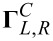 in Equation 6) become ε-independent has a two-fold advantage. This scheme enables one to perform conventional DFT calculations for a finite, isolated, extended molecule (i.e., uncoupled to semi-infinite electrodes). In principle, this can be done with any common DFT package. The implementation is easy, because the post-processing step of adding ε-independent self energies (Equation 1) does not require any DFT-code modification. A further advantage is that the diagonalization can be done before performing transport calculations. This fact drastically reduces the computational effort, as emphasized recently [38]. By definition, the WBL (as well as the FBL) amounts to replacing the exact ε-dependent embedding self-energy with its value at the Fermi energy of the unbiased system, 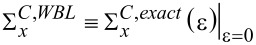. As can be seen from the inspection of Equation 1, Equation 5, and Equation 6, the transmission at the Fermi energy (zero-bias conductance) computed within the WBL coincides with the exact transmission. Therefore,

[33]
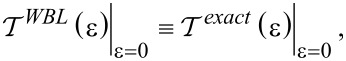


provided that **G***_C_*_,0_ is computed exactly, otherwise

[34]
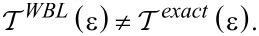


This is a general result that applies to any exact treatment of uncorrelated transport based on the trace formula of Equation 5. Two WBL schemes have been recently utilized within an NEGF-DFT framework [38], termed WBL-Molecule and WBL-Metal; the latter corresponds to a central region including 3 to 6 electrode layers (Figure 4b of [38]). In the present paper, the counterpart of WBL-Molecule is a junction wherein the central region has the Hamiltonian **H** (Equation 9), and the counterpart of WBL-Metal is a central region having the Hamiltonian **H_ext_**(*l*_0_, *r*_0_) (Equation 11, 3 ≤ *l*_0_ = *r*_0_ ≤ 6). In both cases, the corresponding Hamiltonians are supplemented with WBL ε-independent embedding self-energies via Equation 1.

It is instructive to inspect the transmissions 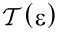 computed at arbitrary energies and *V* = 0 within the full NEGF-DFT (full-SCF in the nomenclature of [38]), the WBL-Molecule, and the WBL-Metal methods shown in Figure 4b of [38].

In the light of Equation 34, the fact that those methods predict transmission values at arbitrary energies 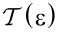 that substantially differ from each other is not at all surprising. An initially surprising point in the present analysis is that (as seen in Figure 4b of [38]) even the transmissions 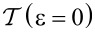 computed at the Fermi energy via the three aforementioned methods do also significantly differ from each other, i.e.,


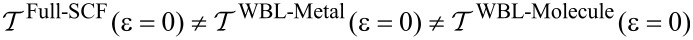

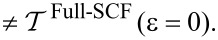


In fact, as expressed by Equation 33, these transmissions should have been equal, i.e.,


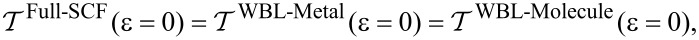


only if all values of **G***_C_*_,0_ were exact. The differences between these values,


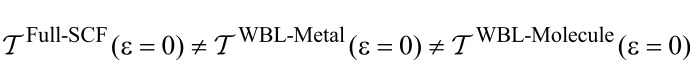

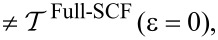


are due to the fact that, unlike the exact model calculation presented in this paper, neither the DFT-method employed to treat the full embedding (full NEGF-DFT), nor that for the isolated molecule (WBL-Molecule), or that for the molecule merely including several electrode layers (WBL-Metal) are exact. Even for a small isolated molecule (here named the WBL-Molecule case), the DFT results represent nothing but more or less (in)accurate approximations. The aforementioned differences also clearly reveal that, even within treatments at the same level of theory (e.g., using the same exchange-correlation functional and basis sets), the results for different molecular sizes are affected by (absolute and relative) errors in a different way. The above analysis also emphasizes that and why, based on “realistic” DFT state-of-the-art transport calculations, it is impossible to demonstrate the invariance envisaged in the present paper. These DFT-based approaches are too inaccurate for this purpose. To eliminate differences resulting from unreliable approaches one should go beyond the DFT level and resort to elaborate many-body schemes [16,39]. These many-body schemes are numerically prohibitive even at the lowest (*GW* [40]) level. For this reason, to be feasible, calculations cannot avoid treating electrodes within WBL(-type) approximations, justifiable only at low energies/biases. From this perspective, the results reported in this paper unfortunately do not convey a very optimistic message. While substantial theoretical improvements within the linear response limit are possible, reliable results for molecular transport beyond the ohmic range cannot be expected from elaborate ab initio many-body approaches (combined with WBL methods) even at (nowadays) numerically completely prohibitive molecular sizes (Figure 5 and Figure 6).

## Conclusion

The results reported in the present paper can be summarized as follows: (i) The independence on the size of the extended molecule used to calculate both the equilibrium and nonequilibrium properties of a nanojunction is a minimal mandatory requirement of any sound theory of molecular transport. It was demonstrated that this invariance property is strictly obeyed for all molecules that can be described within single-particle pictures linked to chain-like electrodes. To the best of our knowledge, the present paper is the first rigorous study and demonstration of this invariance, which is a nontrivial result even for the simplest case of a molecule modeled as a single energy level. Real systems described within this framework include, e.g., atomic chains, quantum wires, carbon nanotubes, and (possibly DNA-based) bio and large organic molecules. To determine the model parameter values, density functional based tight binding (DFTB) frameworks [18–20] represent the state-of-the-art. It is worth emphasizing the generality of the demonstration given in this study. The description of the molecules considered in this paper goes beyond conventional tight-binding nearest-neighbor (extended Hückel) approximations, wherein, for an *N*-site molecular chain, the only nonvanishing matrix elements are the on-site energies *H*_μ,μ_ = α (1 ≤ μ ≤ *N*) and the nearest-neighbor hopping integrals *H*_μ,μ+1_ = *H*_μ+1,μ_ = −β (1 ≤ μ ≤ *N* − 1). Moreover, the Hermitean Hamiltonian matrix **H** of the “physical” molecule does not need to be real (
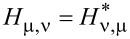
), and the molecule does not need to be one dimensional. Provided that electron correlations are neglected (they are ruled out by the bilinear form of **H** of Equation 10), **H** can include interactions with impurities, applied electric fields (e.g., source–drain bias, gate potentials), static (e.g., Peierls) distortions or proximity to other molecules from the environment. The fact that the matrix elements *H*_μ,ν_ may depend on the bias *V* (e.g., Stark shift of orbital energies) [22,41–43] is particularly noteworthy. (ii) Considering a specific molecule or even a specific class of homologous molecular series, the demonstration of the envisaged invariance property would have been restricted to certain fixed values of the parameters *H*_μ,ν_, possibly exhibiting specific and highly nontrivial bias dependence. From this perspective, it is important to re-emphasize that the invariance demonstrated in this paper holds for arbitrary values of the matrix elements *H*_μ,ν_ and for arbitrary dependencies (e.g., on biases) of these matrix elements. Therefore, in particular, it is not limited to some nanojunctions based on certain molecular species. (iii) Further, it was shown that unlike the exact approach, the approximate approaches based on the limits of wide- and flat-electrode bands nonphysically predict nonequilibrium properties that depend on the size of the central region utilized in calculations. In conjunction with these approximations, the effect of negative differential resistance (NDR) was discussed. It was found that, although qualitatively correct, the quantitative treatment of NDR is unsatisfactory within the FBL. Because the quantitative difference between the exact treatment and FBL is the energy dependence of the width functions Γ*_L,R_* (or, equivalently, the density of states at the contacts), this finding can be reframed as an indication that achieving NDR effects stronger than obtained so far [44] may primarily be a problem of contact engineering. (iv) The analysis done in conjunction with the WBL and FBL has made it clear that studies on nonequilibrium transport properties at finite temperatures require more elaborated theoretical levels than studies on linear response (*V*→0) properties at zero temperature. Low-temperature properties like the low bias conductance (and therefore also the related β factor of exponential attenuation with increasing molecular size) as well as other properties (e.g., thermopower 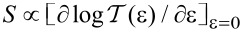
[45]), to which only energies very close to the equilibrium Fermi level contribute, can be obtained within the WBL/FBL. However, studies on the properties away from equilibrium (besides *I*–*V* curves one can also mention current noise power [46–48], for which the knowledge of transmission at the Fermi energy does not suffice [49]) should go beyond the WBL or FBL. (v) Last but not least, it was demonstrated (Figure 2b,c, and Figure 5a,b) that transport calculations with an exact treatment of electrodes adjacent to the active molecule and semi-infinite electrodes within the currently employed WBL or FBL may yield spurious “predictions” of unphysical features in theoretically calculated *I*–*V* curves at higher biases. This procedure, which is equivalent to treating electrode layers included in the extended molecule and semi-infinite electrodes at different levels of theory, resembles current procedures used in “realistic” NEGF-DFT approaches. Therefore, these findings deserve consideration in molecular transport studies beyond the ohmic regime.

## Supporting Information

File 1 Mathematical details for the demonstration that the small molecule and minimally extended molecule yield identical physical properties.
