# Supplementary material for: Invariance of molecular charge transport upon changes of extended molecule size and several related issues
Source: Beilstein J Nanotechnol. 2016 Mar 11;7:418–31. doi: 10.3762/bjnano.7.37 (PMC4901537; doi:10.3762/bjnano.7.37)
Supplement: File 1 — Mathematical details for the demonstration that the small molecule and minimally extended molecule yield identical physical properties. [file Beilstein_J_Nanotechnol-07-418-s001.pdf]

Supporting Information

for

Invariance of molecular charge transport upon changes of extended molecule size and several related issues

Ioan Bâldea[1,2]

Address: [1]Theoretische Chemie, Universität Heidelberg, INF 229, 69120 Heidelberg, Germany and Institute of Space Sciences, National Institute for Lasers, Plasma, and [2]Radiation Physics, National Institute for Lasers, Plasma, and Radiation Physics, 077125, Bucharest-Măgurele, Romania

Email: Ioan Bâldea - ioan.baldea@pci.uni-heidelberg.de

* Corresponding author

Mathematical details for the demonstration that the small molecule and minimally extended molecule yield identical physical properties

To deduce the relationship between the matrix elements of the Green's functions, it may be useful to remember the following determinants' splitting property:

$$\begin{vmatrix} X_{1,1}+Y_{1,1} & X_{1,2} & \dots & X_{1,M} \\ \dots & \dots & \dots & \dots \\ X_{M,1}+Y_{M,1} & X_{M,2} & \dots & X_{M,M} \end{vmatrix} = \begin{vmatrix} X_{1,1} & X_{1,2} & \dots & X_{1,M} \\ \dots & \dots & \dots & \dots \\ X_{M,1} & X_{M,2} & \dots & X_{M,M} \end{vmatrix} + \begin{vmatrix} Y_{1,1} & X_{1,1} & \dots & X_{1,M} \\ \dots & \dots & \dots & \dots \\ Y_{M,1} & X_{M,2} & \dots & X_{M,M} \end{vmatrix} \tag{S1}$$

Notice that the Y values only enter the first column of the determinant in the LHS and of the second determinant of the RHS. To compute the retarded Green's function of the small molecule $\mathbf{G}_C \to \mathbf{G}$ via Equation 1, we need to invert the $N \times N$ matrix $\mathbf{Q}_C \to \mathbf{Q}$

$$\mathbf{Q} = \begin{pmatrix} K_{1,1} - \Sigma_L & \dots & \dots \\ \dots & \dots & \dots \\ \dots & \dots & K_{N,N} - \Sigma_R \end{pmatrix}, \tag{S2}$$

where $K_{\mu,\nu} = \varepsilon\delta_{\mu,\nu} - H_{\mu,\nu}$ (cf. Equations 1 and 10 in the main manuscript). Using Equation S1, the determinant of this matrix can be expressed as

$$|\mathbf{Q}| = |\mathbf{K}| - \Sigma_L (1|\mathbf{K}|1) - \Sigma_R [(N|\mathbf{K}|N) - \Sigma_L (1,N|\mathbf{K}|1,N)]. \tag{S3}$$

Above, $|\mathbf{X}|$ stands for the determinant of the matrix $\mathbf{X}$, and $(i,\dots|\mathbf{X}|\, j,\dots)$ denotes the determinant of the matrix obtained by suppressing the row(s) $i,\dots$ and the column(s) $j,\dots$ from $\mathbf{X}$. In addition to the determinant $|\mathbf{Q}|$, to compute the various matrix elements $G_{\mu,\nu}$ of the Green's function by matrix inversion, the minor determinants $(\mu\,|\mathbf{Q}|\,\nu)$ are needed $(1 \le \mu, \nu \le 1)$

$$G_{\mu,\nu} = (-1)^{\mu+\nu} (\mu\,|\mathbf{Q}|\,\nu) \,/\, |\mathbf{Q}|. \tag{S4}$$

For the relevant indices the matrix elements are given below ($2 \leq \mu, \nu \leq N-1; 1 \leq \eta \leq N-1; 2 \leq \xi \leq N$)

$$(1\,|\mathbf{Q}|\,N) = (N\,|\mathbf{Q}|\,1)^* = (1\,|\mathbf{K}|\,N)\,, \tag{S5a}$$

$$(\mu\,|\mathbf{Q}|\,\nu) = (\mu\,|\mathbf{K}|\,\nu) - \Sigma_R\,(\mu,N\,|\mathbf{K}|\,\nu,N) - \Sigma_L\,[(1,\mu|\,\mathbf{K}\,|1,\nu) - \Sigma_R\,(1,\mu,N|\,\mathbf{K}\,|1,\nu,N)]\,, \tag{S5b}$$

$$(\eta|\mathbf{Q}|1) = (\eta\,|\,\mathbf{k}\,|1) - \Sigma_R\,(\eta,N|\,\mathbf{k}\,|1,N)\,, \tag{S5c}$$

$$(\xi|\mathbf{Q}|N) = (\xi|\,\mathbf{K}\,|N) - \Sigma_L\,(1,\xi|\,\mathbf{K}\,|1,N)\,. \tag{S5d}$$

By choosing next the minimally extended molecule as central region, the RHS of the Dyson equation (Equation 1) is the $(N+1)\times(N+1)$ matrix $\overline{\mathbf{Q}}$ having the form (cf. Equations 1 and 12)

$$\overline{\mathbf{Q}} = \begin{pmatrix} K_{1,1}-\Sigma_L & K_{1,2} & \dots & \dots & 0 \\ K_{2,1} & K_{2,2} & \dots & K_{2,N} & 0 \\ \dots & \dots & \dots & \dots & 0 \\ K_{N,1} & K_{N,2} & \dots & K_{N,N} & \tau_R \\ 0 & 0 & \dots & \tau_R^* & z_R - \overline{\Sigma}_R \end{pmatrix}. \tag{S6}$$

Its determinant $|\overline{\mathbf{Q}}|$ is obtained of the form

$$|\overline{\mathbf{Q}}| = \left(z_R - \overline{\Sigma}_R\right)\left[|\mathbf{K}| - \Sigma_L\,(1|\mathbf{K}|1)\right] - |\tau_R|^2\left[(N|\mathbf{K}|N) - \Sigma_L\,(1,N|\mathbf{K}|1,N)\right]. \tag{S7}$$

It enters the denominator of the corresponding retarded Green's function $\overline{\mathbf{G}} = \left(\overline{G}_{j,k}\right)$ ($1 \leq j,k \leq N+1$)

$$\overline{G}_{j,k} = (-1)^{j+k}\left(j\,|\overline{\mathbf{Q}}|\,k\right)/\,|\overline{\mathbf{Q}}|\,. \tag{S8}$$

The corresponding minor determinants $\left(j\left|\overline{\mathbf{Q}}\right|k\right)$ for the indices of interest are given below for $2 \leq \mu, \nu \leq N-1; 2 \leq \xi \leq N; 1 \leq \eta \leq N-1$:

$$\left(1\left|\overline{\mathbf{Q}}\right|N+1\right) = \left(N+1\left|\overline{\mathbf{Q}}\right|1\right)^* = \tau_R^*\left(1\,|\mathbf{K}|N\right), \tag{S9a}$$

$$\begin{aligned}\left(\mu\left|\overline{\mathbf{Q}}\right|\nu\right) = & \left(z_R-\overline{\Sigma}_R\right)\left[(\mu|\mathbf{K}|\nu)-\Sigma_L\left(\mu,1|\mathbf{K}|\nu,1\right)\right] \\ & -|\tau_R|^2\left[(N,\mu|\mathbf{K}|N,\nu)-\Sigma_L\left(1,\mu,N|\mathbf{k}|1,\nu,N\right)\right],\end{aligned} \tag{S9b}$$

$$\left(\eta|\overline{\mathbf{Q}}|1\right) = \left(z_R-\overline{\Sigma}_R\right)\left(\eta\,|\,\mathbf{K}\,|1\right)-|\tau_R|^2\left(\eta,N|\,\mathbf{K}\,|1,N\right), \tag{S9c}$$

$$\left(N|\overline{\mathbf{Q}}|1\right) = \left(z_R-\overline{\Sigma}_R\right)\left(N|\,\mathbf{K}\,|1\right), \tag{S9d}$$

$$\left(\xi|\overline{\mathbf{Q}}|N+1\right) = \tau_R^*\left[(\xi\,|\,\mathbf{K}\,|N)-\Sigma_L\left(1,\xi\,|\,\mathbf{K}\,|1,N\right)\right], \tag{S9e}$$

$$\left(N|\overline{\mathbf{Q}}|N\right) = \left(z_R-\overline{\Sigma}_R\right)\left(N|\mathbf{K}|N\right)-\Sigma_L\left(1,N\,|\mathbf{K}|\,1,N\right). \tag{S9f}$$

Using the explicit form of the embedding self-energies of Equations 14 and 15, one can easily deduce the following identities

$$\Sigma_R(z_R) = \frac{|\tau_R|^2}{z_R-\overline{\Sigma}_R(z_R)}, \tag{S10}$$

$$w(z_R) \equiv \frac{z_R-\overline{\Sigma}_R(z_R)}{|t_R|} = \frac{z_R}{2|t_R|}+i\sqrt{1-\left(\frac{z_R}{2|t_R|}\right)^2}. \tag{S11}$$

Importantly, $|w(z_R)| = 1$. In view of Equation S10, one gets from Equation S3 and Equation S7

$$\frac{|\overline{\mathbf{Q}}|}{|\mathbf{Q}|} = z_R-\overline{\Sigma}_R = |t_R|w. \tag{S12}$$

Equations S5c and S9c ($1 \leq \eta \leq N-1$), Equations S5b and S9b ($2 \leq \mu, \nu \leq N-1$), and Equations S5d and S9f show that the ratio listed below are equal to the ratio of the determinants of Equation S12

$$\frac{(\eta|\overline{\mathbf{Q}}|1)}{(\eta|\mathbf{Q}|1)} = z_R - \overline{\Sigma}_R = |t_R|w \tag{S13a}$$

$$\frac{(\mu|\overline{\mathbf{Q}}|\nu)}{(\mu|\mathbf{Q}|\nu)} = z_R - \overline{\Sigma}_R = |t_R|w \tag{S13b}$$

$$\frac{(N|\overline{\mathbf{Q}}|N)}{(N|\mathbf{Q}|N)} = z_R - \overline{\Sigma}_R = |t_R|w. \tag{S13c}$$

Equations S5d and S9e and Equations (S5a) and S9a yield ($1 \leq \xi \leq N$)

$$\frac{(\xi|\overline{\mathbf{Q}}|N+1)}{(\xi|\mathbf{Q}|N)} = \tau_R^*. \tag{S14}$$
